# Differential gene expression by lithium chloride induction of adipose-derived stem cells into neural phenotype cells

**DOI:** 10.22038/ijbms.2020.41582.9820

**Published:** 2020-04

**Authors:** Samaneh Farrokhfar, Taki Tiraihi, Mansoureh Movahedin, Hossein Azizi

**Affiliations:** 1 Department of Anatomy, Faculty of Medical Sciences, Tarbiat Modares University, Tehran, Iran; 2 Department of Anatomy, Faculty of Medical Sciences, Tarbiat Modares University, P.O.BOX.14115-331 Tehran, Iran; 3 Department of Physiology, Faculty of Medical Sciences, Tarbiat Modares University, P.O.BOX.14115-331 Tehran, Iran

**Keywords:** Induction, Lithium chloride, Neuron-like cells, Stem cells, Transdifferentiation

## Abstract

**Objective(s):**

Adipose-derived stem cells (ADSCs), with suitable and easy access, are multipotential cells that have the ability for differentiation into other mesodermal and transdifferentiate into neural phenotype cells. In this study, Lithium chloride (LiCl) was used for *in vitro* transdifferentiation of rat ADSCs into neuron-like cells (NLCs).

**Materials and Methods:**

ADSCs were isolated from the rats’ perinephric region using Dulbecco΄s Modified Eagle΄s Medium (DMEM) with Fetal Bovine Serum (FBS), cultured for 3 passages, characterized by flowcytometry and differentiation into adipogenic and osteogenic phenotypes. The ADSCs were exposed to 0.1, 0.5, 1, 1.5, 2, 5, and 10 millimolar (mM) LiCl without serum for 24 hr. The optimum dose of LiCl was selected according the maximum viability of cells. The expression of neurofilament light chain (NfL), neurofilament high chain (NfH), and nestin was evaluated by immunocytochemistry. Quantitative reverse transcription polymerase chain reaction (qRT-PCR) was used to evaluate the amount of synaptophysin, neurogenin-1, neuroD1, NfL, NfH, and nestin genes’ expression in ADSCs and NLCs.

**Results:**

The optimum dose of LiCl was 1 mM in 24 hr. The transdifferentiated ADSCs showed cytoplasmic extension with synapse-like formation. Synaptophysin, neurogenin-1, neuroD1, NfL, NfH, and nestin genes were significantly expressed more in NLCs than in ADSCs.

**Conclusion:**

LiCl can induce ADSCs into neural phenotype cells with higher expression of neural and neuronal genes.

## Introduction

Cell therapy is a feasible approach for treating neurological disorders when adult stem cells in the nervous system cannot produce appropriate response for neuronal loss. In other words, cell therapy is considered a mode of replacement therapy (1). Stem cells with the ability of replication and differentiation are used for treatment of many disorders and abnormalities in various organs of the body such as the nervous system. After birth, some cells, like neural cells; cannot proliferate (1). Therefore, during cell therapy, other stem cells should be used to repair neural diseases and injuries. On the other hand, in most treatment processes, time is an important factor. So, it is important to select an appropriate cell source with a safe and nontoxic inducer that can be differentiated in the shortest time possible. 

One of the strategies for choosing the source stem cells is to select the cells from the tissues that can be easily accessed with low risk (2), such as adipose-derived stem cells (ADSCs) (3). They are multipotent stem cells, which can differentiate into other mesodermal lineages (4). On the other hand, it has been shown that ADSCs can transdifferentiate into other germ lines like neural cells and glial cells (3) to be used for repairing neural lesions including spinal cord injury in rats (3).

The process of transdifferentiation requires neural inducers such as dimethyl sulfoxide and retinoic acid for differentiating stem cells into neural phenotype cells (5, 6). However, some of these substances are toxic and cannot be used in clinical practice (7, 8). Lithium chloride (LiCl) is widely used in clinical neurology in the treatment of neurological diseases including bipolar disease, Alzheimer’s diseases, Parkinson’s disease, and spinal cord injury. It is also effective in differentiation of stem cells into neural phenotype (9-16). Moreover, it has a neuroprotective effect by increasing the brain-derived neurotrophic factors, vascular endothelial growth factor expression, anti-apoptotic effects through up-regulation of B-cell lymphoma protein-2 (Bcl-2) protein, suppressing the calcium-dependent activation of pro-apoptotic signaling pathways (10, 17, 18), inhibiting glycogen synthase kinase 3β, mimicking the effect of Wnt signaling on gene expression, as well as cellular proliferation and differentiation (10, 18). LiCl inhibits apoptosis through P53 and Bax signaling (19). It has been shown that after 24 hr, 0.5 mM LiCl is able to induce rat bone marrow cells into cells with neural phenotype (20). 

In the present study, ADSCs were induced into neuron-like cells (NLCs) by LiCl and the level of neural and neuronal genes was quantitatively evaluated using quantitative reverse transcription polymerase chain reaction (qRT-PCR).

## Materials and Methods

***Isolation of ADSCs***

ADSCs were isolated from the perinephric region of Sprague-Dawley rats with the weight of approximately 250–300 g (Pasteur Institute of Iran, Tehran, Iran). The work was approved in the Ethical Committee of Tarbiat Modares University (Tehran, Iran) based on Helsinki ethical code. The animals were completely anesthetized with chloroform and their perinephric region was shaved and treated with betadine. An incision was made, the adipose tissue was exposed, and under sterile conditions, the adipose tissue was sampled. Then it was transferred to a Falcon tube containing sterile phosphate buffer saline (PBS) with Penicillin 100 IU and Streptomycin 100 mg per ml (penicillin/streptomycin). Next, fresh adipose tissue was washed several times with sterile PBS to remove blood contaminations. Then the tissue was cut with a sterile surgical knife and incubated with 0.075% collagenase type 1 (equivalent to the volume of tissue) for 30 min in a 37 ^°^C incubator with gentle shaking and pipetting. Finally, a milky solution was obtained and the enzyme was neutralized with a medium containing 10% fetal bovine serum (FBS). Then it was centrifuged at about 1800 RPM for 10 min. The supernatant was discarded and the cells at the bottom were cultured in a flask containing Dulbecco΄s Modified Eagle΄s Medium (DMEM) medium (supplemented with penicillin/streptomycin and 10% FBS), which was placed in a 37 ^°^C humidified incubator with 5% CO_2_ for 5 days. When the confluency reached about 70 ~ 80%, the cells were washed three times with PBS, harvested with trypsin and ethylenediaminetetraacetic acid (EDTA) solution, centrifuged and cultured. This process continued until passage 3 (21, 22).

***Characterization of the isolated cells***

The ADSCs were evaluated for adipogenesis and osteogenesis where the cells at the third passage were cultured in two plates, one having adipogenic differentiation medium (containing 50 μg/ml indomethacin, 50 μg/ml ascorbic acid, and 100 nM dexamethasone), and the other having osteogenic differentiation medium (containing 10 mM β-glycerophosphate, 60 μM ascorbic acid, and 0.1 μM dexamethasone) for 21 days. The adipogenic and osteogenic differentiation of ADSCs was confirmed with Oil Red O and Alizarin Red S, respectively. On the other hand, the surface markers were evaluated with flowcytometry using CD90, CD44, CD73 (positive markers), and CD34 (hematopoietic marker) (22). This experiment was repeated three times.

***Evaluation of the viability of ADSCs***

Trypan blue exclusion test was done to evaluate the viability of ADSCs. The cells were suspended in PBS, a sample was stained with 0.4% Trypan blue (equal volume), and then their viability was estimated with Neubauer chamber under a light microscope. The viability was 97%.

***Determination of LiCl’ optimal dose***

Determination of the optimal dose of LiCl (Sigma, Germany) for induction was performed using 0.1, 0.5, 1, 1.5, 2, 5, and 10 mM of LiCl (20). The cells were seeded at the third passage with these doses in DMEM medium without FBS for 24 hr. Then the cell viability in each dose was estimated and compared with the positive control (ADSCs in DMEM with FBS) and negative control (ADSCs in DMEM without FBS). This experiment was repeated three times.

***Identification of cells after induction***

The LiCl-induced ADSCs were evaluated morphologically using an invert microscope. The differentiation was checked by immunostaining with neurofilament light chain (NfL) (Millipore, UK,1:100), neurofilament heavy chain (NfH) (Millipore, UK,1:100), and nestin (Abcam, UK,1:100). The cells were seeded on a cover slip for 24 hr, and cultured in DMEM containing LiCl (1 mM) for 24 hr. Then the cells were fixed with 4% paraformaldehyde. After treatment with the blocking solution, the cells were immunostained with primary antibodies, and finally, labeled with the secondary antibodies. 

***Quantitative expression of neural and neuronal genes***

The RNA of the induced cells with LiCl was extracted using (Qiagen, Germany). Then the cDNA was synthesized using Thermo kit, UK. Next, the level of synaptophysin, neurogenin-1, neuroD1, NfL, NfH, and nestin genes’ expression in NLCs and ADSCs was determined using qRT-PCR and then their relative gene expression was estimated. GAPDH was also used as an internal control (housekeeping gene) (23, 24). The experiments were done in triplicate. The sequence of primers is given in Table 1. 20 µl of the samples was analyzed for each group; then 10 µl of the products was taken for qualitative evaluation on a gel. The length of the products was determined by electrophoresis and their identity was confirmed.

## Results

**Figure 1:**
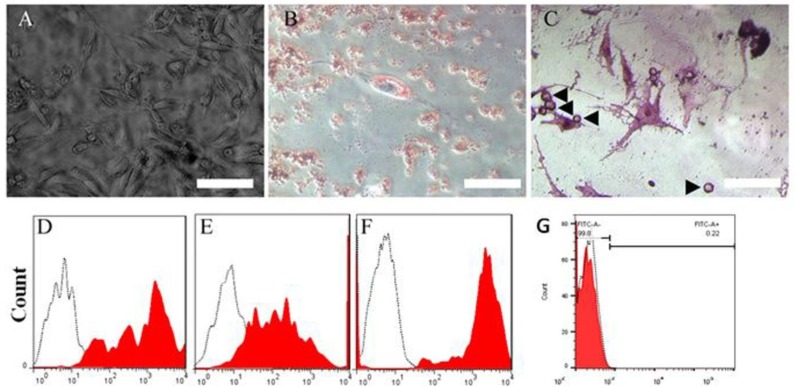
Morphology and characterization of the isolated adipose-derived stem cells (ADSCs)

**Figure 2:**
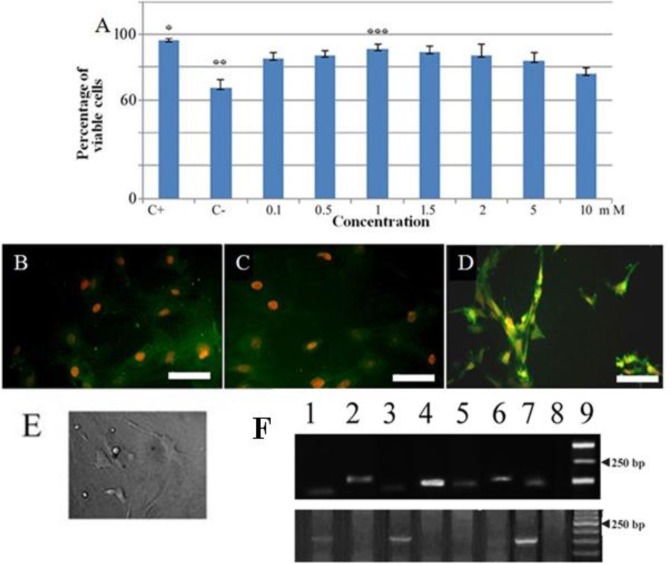
A) Dose-response evaluation of Lithium Chloride (LiCl) using the viability of the induced adipose-derived stem cells (ADSCs) by LiCl. Single asterisk indicates significantly higher than the other groups (*P-value<*0.05). Double asterisks indicate lower than the other groups (*P-value<*0.05). Triple asterisks indicate 1 mM is significantly higher than 10 mM concentration (*P-value<*0.05), while the difference with other groups was insignificant. B, C, and D show immunostaining with nestin, neurofilament light chain (NfL) and neurofilament heavy chain (NfH), respectively (Scale bar: B, C, and D=100 µm). E shows the phase contrast image of the neuron-like cells transdifferentiated from ADSCs with neuronal morphology (Scale bar=800 µm). F demonstrates the reverse transcription polymerase chain reaction (RT-PCR) of the isolated RNA from the neuron-like stem cells (upper panel). 1-9: NfL, neuroD1, nestin, neurogenin-1, synaptophysin, NfH, GAPDH (housekeeping gene), no template control (NTC) and DNA ladder, respectively; lower panel represents the electrophorogram of RT-PCR from RNA isolated from adipose-derived stem cells

**Figure 3:**
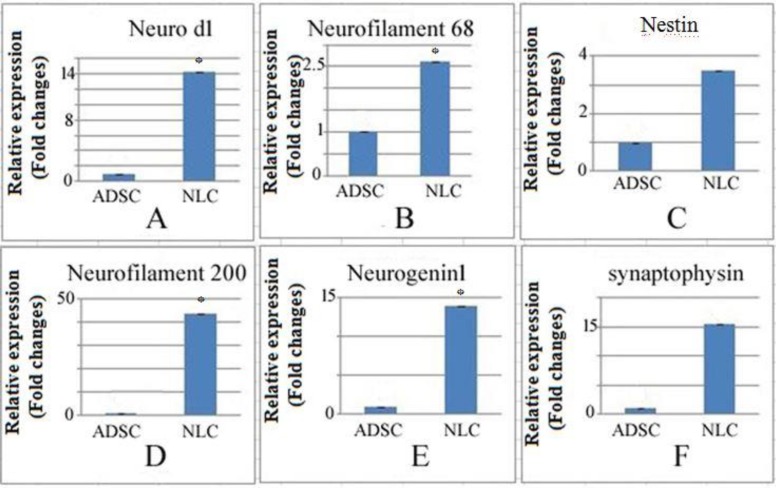
Quantitative expression using quantitative reverse transcription polymerase chain reaction (qRT-PCR) of neuroD1 (A), neurofilament light chain (68), or NFL(B), nestin(C), neurofilament heavy chain (200) or NfH (D), neurogenin-1 (E) and synaptophysin (F). Asterisk indicates significantly higher than the external control (*P-value<*0.05)

The isolated cells’ morphology is presented in the phase contrast image (Figure 1A). The lipogenesic and osteogenesic differentiation of ADSCs are demonstrated in Figures 1B and C, respectively. The markers of ADSCs are presented in Figure 1 D-G), for CD90, CD44, CD73, and CD34 (97%, 99.5%, 99.9%, and 2%, respectively). The viability of ADSCs at passage 3 using trypan blue exclusion assay was 97%±3.5. The dose-response of LiCl is presented in Figure 2A, statistical analysis shows using the viability for evaluation of the optimal dose, the positive control was significantly higher than the other groups (*P*<0.05); in the LiCl-treated groups, it was significantly higher than in the negative control one (*P*<0.05). The highest viability was noticed in the dose of 1 mM (90.83±2.99), which was significantly higher than 10 mM, while the differences among other concentrations were not significant. After 24 hr of induction of the cells by LiCl, using phase contrast microscopy, the induced ADSCs showed extension with bipolar or multipolar morphology, and formed connections with other cells by axon-like structure (Figure 2E). Immunostaining of these cells with neural and neuronal markers (nestin, NfL, NfH) indicated the transdifferentiation of ADSCs into NLCs (Figures 2 B-D). In RT-PCR profile, NLCs showed that NfL, neuroD1, neurogenin-1, and NfH were expressed while synaptophysin and nestin genes were not expressed. On the other hand, the profile of ADSCs showed the expression of NfL and nestin only. The qRT-PCR outcome showed that the expression of all genes, increased significantly comparing to the control group, except for nestin and synaptophysin (*P*<0.05). An increase was seen in the expression of neurogenin-1 and neuroD1, while the lowest expression was in nestin and NfL. NfH was fairly expressed. The qRT-PCR results are presented in Figure 3. NfH showed the highest expression level (44-fold increase in NLCs as compared with ADSCs), while synaptophysin, neuroD1, neurogenin-1, nestin, and NfL had 16, 14, 13.5, 3.5, and 2.7-fold increase, respectively. The primers are listed in Table 1.

## Discussion

The purpose of this study was to investigate the effect of LiCl on transdifferentiation of ADSCs into NLCs at 1 mM dose incubated for 24 hr. The transdifferentiation was confirmed by morphology, immunocytochemistry, and qRT-PCR. These results are consistent with the findings of others (20, 25). As mentioned, LiCl could induce the expression of synaptophysin, neurogenin-1, neuroD1, NfL, NfH, and nestin; however, the level of expressions was significant except in nestin and synaptophysin it was insignificant. Each of these genes is expressed in different stages of the development of the nervous system. Nestin is a type IV intermediate filament protein expressed in neural stem/progenitor cells and shows early neural differentiation (26-28). Neuroblastoma cell types can express nestin which is considered a stemness marker (27, 28). It is believed that in neural development, nestin is replaced by neurofilaments (Nfs). This replacement can be effective in the change of cellular shape (28). NeuroD1 and neurogenin-1 are in the bHLH (basic helix–loop–helix) transcription factors’ gene group, which are necessary for the development of many types of neurons in the nervous system (29). They are expressed during the development of the nervous system and regulate neurogenesis, differentiation and/or gliogenesis (25-27). NeuroD1 is expressed in immature neurons (27). Neurogenin-1 is expressed earlier than neuroD1 (30, 31). Ross *et al*. reported that bHLH motifs modulate critical events in the development of the mammalian neocortex where neurogenin-1 is involved in transition from proliferative phase to neurogenesis phase (32), resulting in cell fate determination (33). Researchers reported that neuroD1 overexpression is involved in regulation of neuronal migration (34). Also overexpression of neuroD1 promotes the conversion of glial cells into neurons (35). Human embryonic stem cells could be induced into neuronal phenotype cells by overexpression of either neurogenin-1 or neuroD1 (36). Similar results were documented with human pluripotent stem cells (37). Khalfallah *et al*. documented that the simultaneous up-regulation of neurogenin-1 and neuroD1 could lead to an accelerated neuronal differentiation (38). The expression of NfL and nestin in ADSCs was noticed in this investigation, which can be due to heterogeneity of the mesenchyme derived stem cells (39), Mo *et al*. reported that nestin expressing mesenchymal stem cells may result from isolation and cultivation regimes (40).

Nfs are intermediate and main cytoskeletal proteins in mature neurons that contribute to neuronal development and function, though NfH usually express after NfL (41, 42). Their main role is to enhance the axonal caliber of myelinated axons, and thus, increase the axonal conduction velocity (42). Loss of NfL occurs in small caliber axons (26). Synaptophysin is a presynaptic protein and an essential for endocytosis, docking, fusion, and membrane trafficking in presynaptic site in mature (at both excitatory and inhibitory synapses) neurons (42-44). The amount of synaptophysin expression is age-related, and its peak is between 5-10 years (45). So, it can be said the order of their expression in developmental stages is as follows: nestin, neurogenin-1, neuroD1, NfL, NfH, and synaptophysin, which sometimes have synchronization (46).

In this study, the morphological, immunocytochemical, and molecular evaluations confirmed the transdifferentiation of ADSCs into NLCs. However, the qRT-PCR results showed increased expression in all genes but nestin, as a gene of the early stages of development and differentiation, and synaptophysin, as a gene of the late stages of development and differentiation had no significant difference compared with the control group. Also it seems that given the great difference in the expression of NfH compared with the ADSCs group and the highest difference compared with other genes that were significant, it seems that these cells have reached maturity, but they are not yet functional. So, it can be said that this transdifferentiation has proceeded for post-mitotic neurons; however, the process of differentiation has not yet been fully achieved, and may require more incubation time with LiCl or secondary inducer to accelerate the post-mitotic neuron differentiation. Of course, whether these cells are functional cells or not, will require further investigation. Also it is important to mention that although the viability of the positive control was significantly higher than that of the other groups, it was significantly higher in the LiCl-treated groups than in the negative control group, so it can be due to the addition of FBS to the positive control only, while the treatment groups and negative control were deprived of FBS. Moreover, FBS is required for cell survival, LiCl is able to increase viability with the negative control. FBS can increase cell proliferation. The reason that it affects cell viability seems to be that it can affect normal mitochondrial function, hold the amount of mitochondrial protein, and retain the balance of the gene expression associated with oxidant. On the other hand, it prevents the aggregation of ROS and suppresses the expression of P53, P51, and p21; so, it can be said it inhibits apoptosis (47, 48). The current investigation demonstrated that LiCl has a neuroprotective effect, which is similar to the findings of recent studies (18). LiCl, by inhibiting glycogen synthase kinase 3β and mimicking the effects of Wnt signaling on gene expression, cellular proliferation, and differentiation, is effective on the nervous system (10, 18). In addition, lithium exerts its neuroprotective effect by increasing the brain-derived neurotrophic factors, vascular endothelial growth factor expression, anti-apoptotic effects through up-regulation of B-cell lymphoma protein-2 (Bcl-2) protein, and suppressing the calcium-dependent activation of pro-apoptotic signaling pathways (10, 17). It further affects several brain signaling cascades, including increasing the amount of protein G-encoding mRNA and cyclic adenosine monophosphate (10). The important point is that LiCl inhibits apoptosis through P53 and Bax signaling (19). Lithium has been proven to increase the survival of GABAergic neurons (49). Due to the short induced time (24 hr), it seems that LiCl can be a suitable option for neural induction. Apparently, due to the simpler and non-invasive method of preparation and isolation of ADSCs, as well as their more proliferation ability and easier growth than the bone marrow stem cells (4), they can be more useful in regenerative medicine. On the other hand, these cells are heterogeneous (50-52), have good homing in damaged tissue, reduce inflammation, modulate immunity, and release important growth factors to heal wounds (52); all these are good reasons for choosing ADSCs. 

In cell therapy, it is difficult to obtain endogenous neural stem cells because they are located in subventicular and subgranular zones. Induced neural stem cells such as ADSCs are a feasible alternative source, which is a safe and efficient option. On the other hand, LiCl is an inducer that has considerable safety and can induce stem cells in a very short time, which can be helpful in regenerative medicine.

**Table 1:** The name of genes used in reverse transcription polymerase chain reaction (RT-PCR) and quantitative reverse transcription polymerase chain reaction (qRT-PCR), the sequence of forward and reverse primers, the size of fragments, and melting temperature (TM)

Gene	Sequences of forward and reverse primers	Fragment size	TM
Synaptophysin	CCCTACATTCACCCACTTCTCC AACTCACACCAACCCACTCCA	184	60.09 61.53
Neurofilament light chain (NfL or NF68)	AGAAGAAGGTGGTGAGGGTGA TTTACTGGGATAGTTGGGAATGG	132	60.41 57.88
Neurofilament heavy chain (NfH or NF200)	CTTTTGACCCAGTCCCTTCTCTC TGCCTCTTCTTCTTCCTCCCCT	217	60.56 62.53
Nestin	ACTCTCCCTCACTCTACTCCCCT TCCCTATGTCCTCAAACTCTTCA	223	63.05 58.57
Neurogenin-1	CCAACCACAACCTTCCTCAATC ACAGTCCCTCGTCTCCTTCA	188	59.44 59.89
GAPDH	AAGTTCAACGGCACAGTCAAGG CATACTCAGCACCAGCATCACC	121	61.58 61.32
NeuroD1	CTGGAAGAGGAGGAAGAGGAGGA CTGTGTGTTAGAGTAGCAGGGT	210	62.24 59.44

A) phase contrast image of the isolated cells at passage 3. Most of the cells are spindle-shaped similar to fibroblastic cells. B) Adipogenic differentiation of ADSCs following treatment with lipogenic differentiation medium, and staining with Oil Red O, the cells show multilocular fat droplet. C) Osteogenic differentiation of ADSCs; following treatment with osteogenic differentiation medium, and staining with Alizarin Red S, the cells (Arrow head) show ossicle (Scale bar: A-C=100 µm). Figures 1D- G: flowcytometry of the isolated cells (CD90, CD44, CD73, and CD34, respectively)

## Conclusion

LiCl can induce adipose-derive stem cells into neuronal phenotype and expression of neurogenin-1, neuroD1, NfL, and NfH, except nestin and synaptophysin genes were significantly expressed in transdifferentiated cells. Because of the importance of time factor along with selection of available cellular source and appropriate inducer, it seems that using ADSCs with LiCl can be a good choice for curing the nervous system diseases.
